# Predictive model of unfavorable outcomes for multidrug-resistant tuberculosis

**DOI:** 10.11606/s1518-8787.2019053001151

**Published:** 2019-09-12

**Authors:** Luiz Henrique Arroyo, Antônio Carlos Vieira Ramos, Mellina Yamamura, Thais Zamboni Berra, Luana Seles Alves, Aylana de Souza Belchior, Danielle Talita Santos, Josilene Dália Alves, Laura Terenciani Campoy, Marcos Augusto Moraes Arcoverde, Valdes Roberto Bollela, Sidney Bombarda, Carla Nunes, Ricardo Alexandre Arcêncio

**Affiliations:** I Universidade de São Paulo. Escola de Enfermagem de Ribeirão Preto. Ribeirão Preto, SP, Brasil; II Universidade de São Paulo. Faculdade de Medicina de Ribeirão Preto. Ribeirão Preto, SP, Brasil; III Secretaria de Estado da Saúde de São Paulo. São Paulo, SP, Brasil; IV Universidade NOVA de Lisboa. Escola Nacional de Saúde Pública. Lisboa, Portugal

**Keywords:** Tuberculosis, Multidrug-Resistant, complications, Tuberculosis, Multidrug-Resistant, mortality, Risk Factors, Treatment Adherence and Compliance

## Abstract

**OBJECTIVE:**

to analyze the temporal trend, identify the factors related and elaborate a predictive model for unfavorable treatment outcomes for multidrug-resistant tuberculosis (MDR-TB).

**METHODS:**

Retrospective cohort study with all cases diagnosed with MDR-TB between the years 2006 and 2015 in the state of São Paulo. The data were collected from the state system of TB cases notifications (TB-WEB). The temporal trend analyzes of treatment outcomes was performed through the Prais-Winsten analysis. In order to verify the factors related to the unfavorable outcomes, abandonment, death with basic cause TB and treatment failure, the binary logistic regression was used. Pictorial representations of the factors related to treatment outcome and their prognostic capacity through the nomogram were elaborated.

**RESULTS:**

Both abandonment and death have a constant temporal tendency, whereas the failure showed it as decreasing. Regarding the risk factors for such outcomes, using illicit drugs doubled the odds for abandonment and death. Besides that, being diagnosed in emergency units or during hospitalizations was a risk factor for death. On the contrary, having previous multidrug-resistant treatments reduced the odds for the analyzed outcomes by 33%. The nomogram presented a predictive model with 65% accuracy for dropouts, 70% for deaths and 80% for failure.

**CONCLUSIONS:**

The modification of the current model of care is an essential factor for the prevention of unfavorable outcomes. Through predictive models, as presented in this study, it is possible to develop patient-centered actions, considering their risk factors and increasing the chances for cure.

## INTRODUCTION

Multidrug-resistant tuberculosis (MDR-TB) is considered a global public health problem and a major threat to the control and elimination of tuberculosis (TB) in the world. Characterized by a bacillus resistant to isoniazid and rifampicin, two of the main drugs in the initial treatment regimen, it is estimated that MDR-TB in 2016 reported about 490,000 cases, equivalent to 4.7% of the total number of people who became ill from TB in the world^1^.

According to the latest report by the World Health Organization (WHO), 54% of cases started treatment for MDR-TB in 2014, 54% have successfully completed it (cure or complete treatment), 15% have lost to follow-up, 8% had some kind of failure, and 16% died. Comparing the outcomes of new cases or recurrences of TB in which the bacillus is sensitive to first-line drugs, there was an 83% cure, indicating that increasing success rates in MDR-TB treatment is one of the major global challenges for disease control^1^.

The difficulty of success is a consequence of the insufficiency of therapies that allow to coordinate treatments more effective and with greater capacity for the favorable outcome. Treatment regimens are still based on fragile scientific evidence, and drugs used do not always undergo randomized controlled trials, which results in a high frequency of treatment failures^2^.

In addition, long periods of exposure to drugs with high toxicity that can trigger serious adverse effects, coupled with treatments that raise catastrophic household expenditures, social stigma and psychological stress, reduce adherence and tolerance of patients to the MDR-TB treatment and increase the risk for unfavorable outcomes such as death, abandonment and treatment failure^3,4^.

Despite this, the country has low rates of cure for MDR-TB treatment, reaching only 61.4%, a percentage below that established by WHO (75%)^1^. Thus, it is imperative to identify the barriers that prevent the cure of patients, under penalty of the transmission of resistant *Mycobacterium tuberculosis*, which contributes to the increase of the proportion of MDR-TB between incident of TB cases and the development of forms of the disease, such as extensively resistant tuberculosis (XDR-TB)^5^.

Thus, considering the complexity involved in the success of MDR-TB treatment and the lack of research on the subject, this study aims to characterize the outcomes of MDR-TB treatment and to analyze the temporal trend and factors related to unfavorable outcomes in the state of Sao Paulo.

## METHODS

### Study design and population

Retrospective cohort study using secondary data from the state TB case notification system, TBWeb. All cases diagnosed with MDR-TB between the years 2006 and 2015 were used in the 645 municipalities of the state of São Paulo. The data were collected at the Center for Epidemiological Surveillance Prof. Alexandre Vranjac in December 2017.

### Variables and statistical analysis

Initially, in the exploratory approach of the database the duplications were removed. In this process the full name of the individual, full name of the mother and date of birth were used, remaining for the analyzes only the most current outcome.

The descriptive analysis considered the number of previous treatments for MDR-TB and the individual characteristics present in TBWeb’s case report form: sociodemographic (gender, age, ethnicity, schooling and type of address), clinical (clinical form of MDR-TB and associated comorbidities) and operational (way of discovering the case and supervised or self-administered treatment). It is worth noting that unfavorable outcomes were those cases whose last closure was death, abandonment and treatment failure.

The unfavorable outcomes in the treatment of MDR-TB are established by the National Program for the Control of Tuberculosis (PNCTB) as treatments that evolve negatively and result in dropouts, treatment failure or deaths. Abandonment is defined as not taking the medication for more than 30 consecutive days. Treatment failure is defined by two or more positive cultures after the 12th month of treatment or according to the assessment of the patients’ clinical status^6^.

Then, the annual occurrence of each outcome was verified, including all types available for completion in the notification form (cure, abandonment, treatment failure, TB death, non-TB death, other outcomes, without outcome information). Subsequently, analyzes of temporal trends (temporal regression), whose predictive variables were the number of cure outcomes, abandonment, treatment failure, death with basic TB cause and death without basic TB cause, and the response variable was time (in years).

The outcomes were logarithmized, reducing the heterogeneity of the residue variance in the time regression. This time trend was performed using the self-reported analysis method known as Prais-Winsten, whose result is called the annual increase rate, with a 95% confidence interval (95%CI). Significant results could represent the annual increase or decrease in the occurrence of outcomes, while non-significant outcomes may be considered stationary^7^.

To verify the factors related to the unfavorable outcomes, binary logistic regression was used, having as reference the treatments with cure, and as independent variables the individual information of the patients. It should be emphasized that different models were used for each of the outcomes.

In the first step, the crude odds ratio (OR) with 95%CI was calculated. Subsequently, the variables with significant OR were included in the multiple model with the forward method (likelihood ratio), determining their adjusted odds ratio (ORaj). For both final models, the determination of pseudocofficients (McFadden R^2^), Wald statistic and prediction or accuracy of the models were calculated using the area below the characteristic curve of the receiver operating characteristic (ROC) and its values of 95%CI. The values of the ROC curve were interpreted according to Šimundić^8^.

Pictorial representations of the factors related to death, abandonment and failure in the treatment of MDR-TB and its prognostic capacity for the outcomes in the form of probabilities were made. Such a technique is called nomogram and is presented as a scoring scale for each variable introduced in the analysis. This score is equivalent to a certain probability for the event being studied, in this case, treatment outcomes^9^.

### Ethical Aspects

The study was approved by the Research Ethics Committee of the University of São Paulo at School of Nursing of Ribeirão Preto on September 12, 2017, with an Ethics Presentation Certificate (CAAE) protocol number 71051017.8.0000.5393.

## RESULTS

A total of 1,168 MDR-TB reports were identified in the state of São Paulo from 2006 to 2015. After filtering the duplicate reports, the total number of 802 patients affected by the disease was verified. Their sociodemographic and clinical-operational characteristics are presented in Table 1.

**Table 1:** Profile of 802 cases of multidrug-resistant tuberculosis reported in the state of São Paulo from 2006 to 2015.

Variable	n		%
Outcome of treatments			
Cure	323		40.3
Abandonment	66		8.2
Treatment failure	275		34.3
Change of diagnosis	6		0.7
Non-tuberculosis death	55		6.8
Death due to tuberculosis	56		7.0
Transfer	5		0.7
No information	16		2.0
No. of treatments			
One treatment	522		65.1
More than one treatment	280		34.9
Ethnicity			
White	358		44.6
Black/brown	267		33.3
Others (yellow, indigenous)	6		0.7
No information	171		21.3
Age (years old)			
≤ 14	7		0.9
15–29	197		24.5
30–59	532		66.3
≥ 60	66		8.2
Gender			
Male	564		70.3
Female	238		29.7
Education level			
≤ 7 years	354		44.1
> 7 years	311		38.8
No information	137		17.1
Clinical Form			
Pulmonary	782		97.5
Extrapulmonary	20		2.5
Way of discovering			
Outpatient demand	531		66.2
Urgency/emergency or during hospital stay	205		25.6
Active case search	38		4.7
No information	28		3.5
HIV Testing			
Executed	735		91.6
Unexecuted	67		8.4
AIDS			
Yes	104		13.0
No	621		77.4
No information*	77		9.6
Diabetes			
Yes	103		12.8
No	699		87.2
Alcoholism			
Yes	183		22.8
No	619		77.2
Mental illness			
Yes	12		1.5
No	790		98.5
Use of illicit drugs			
Yes	96		12.0
No	706		88.0
Smoking			
Yes	70		8.7
No	732		91.3
Type of address			
Default Address	742		92.5
Prisoner	44		5.5
Without fixed residence	16		3.0
Treatment type			
Supervised	663		76.5
Self-administered		154	17.7
No Information		50	5.8

* 10 cases with HIV positive test but not classified with AIDS were considered without information.

Cure was the treatment outcome with the highest prevalence (n = 323, 40.3%); however, treatment failure was almost as frequent, with 275 (34.3%) occurrences. Other unfavorable outcomes, such as abandonment and death with a basic TB cause, occurred in 15.2% (n = 122) of the cases. Despite the predominance of patients undergoing only one treatment, 34.9% (n = 280) had previously been treated for MDR-TB.

Regarding the sociodemographic profile, there were predominant cases of males, aged between 15 and 59 years, white and with less than seven years of schooling. Concerning the clinical-operational characteristics, the pulmonary form of the disease prevailed and the diagnosis was made in general by outpatient demands; however, a quarter of the cases were found in emergency services or during hospital stay (n = 205, 25.6%).

None of the registered comorbidities showed to be more prevalent among the cases; however, the most frequent comorbidity was alcoholism, followed by HIV/AIDS and diabetes. Most of the registered patients had a standard address (n = 742, 92.5%), with individuals deprived of freedom forming 5.5% (n = 44) of the cohort and the remainder representing persons without a fixed residence.

Observing treatment outcomes per year studied (Table 2), it can be seen that the average cure rate between 2006 and 2010 was 25.77% and 55.22% in subsequent years. Time trend analysis demonstrated the growth rate of cure at 5% per year. Failure in treatment was the predominant outcome among the first four years studied; however, after 2011 this percentage dropped, reaching only 7.29% in 2015. In the interpretation of the time trend, this outcome decreased around 8% per year. In addition, TB abandonment and deaths remained practically constant in the observed years, which was confirmed by the steady tendency of the cases.

**Table 2:** Distribution of treatment outcomes by year and temporal trend, São Paulo, 2006-2015.

Outcome	2006	2007	2008	2009	2010	2011	2012	2013	2014	2015	Total	Coefficient	95%CI	Temporal trend
									
	n (%)	n (%)	n (%)	n (%)	n (%)	n (%)	n (%)	n (%)	n (%)	n (%)				
Cure	28 (34.57)	18 (26.87)	20 (29.85)	14 (14.58)	20.00 (22.99)	47 (50.54)	42 (54.55)	33 (55.93)	44 (55.70)	57 (59.38)	323 (40.27)	0.05	0.01–0.09	Increasing
Abandonment	4 (4.94)	4 (5.97)	7 (10.45)	12 (12.50)	5.00 (5.75)	4 (4.30)	5 (6.49)	6 (10.17)	7 (8.86)	12 (12.50)	66 (8.23)	0.03	-0.02–0.08	Stationary
Treatment failure	37 (45.68)	34 (50.75)	33 (49.25)	52 (54.17)	42.00 (48.28)	30 (32.26)	17 (22.08)	12 (20.34)	11 (13.92)	7 (7.29)	275 (34.29)	-0.08	-0.01– -0.02	Decreasing
Death TB	3 (3.70)	7 (10.45)	1 (1.49)	8 (8.33)	8 (9.19)	4 (4.30)	5 (6.49)	5 (8.47)	7 (8.86)	8 (8.33)	56 (6.98)	0.03	-0.01–0.08	Stationary
Death not TB	6 (7.41)	2 (2.98)	4 (5.97)	9 (9.37)	10 (11.49)	5 (5.38)	7 (9.09)	3 (5.08)	6 (7.59)	3 (3.12)	55 (6.86)	-0.01	-0.06–0.05	Stationary
Other	2 (2.47)	1 (1.49)	0 (0)	0 (0.00)	2 (2.30)	2 (2.15)	1 (1.30)	0 (0)	1 (1.27)	2 (2.08)	11 (1.38)	-	-	-
No Information	1 (1.23)	1 (1.49)	2 (2.99)	1 (1.04)	0 (0)	1 (1.08)	0 (0.00)	0 (0)	3 (3.80)	7 (7.29)	16 (1.99)	-	-	-

Total	81 (10.10)	67 (8.35)	67 (8.35)	96 (11.97)	87 (10.85)	93 (11.60)	77 (9.60)	59 (7.36)	79 (9.85)	96 (11.97)	802 (100)	<0.01	-0.01–0.02	Stationary

TB: tuberculosis

In the analysis of the factors associated with unfavorable outcomes in the studied cohort (Table 3), the crude OR values adjusted for death with basic TB cause, abandonment and treatment failure are presented. The history of one or more previous treatments of MDR-TB (ORaj = 0.33, 95%CI 0.16-0.66) was a protective factor for abandonment, while illicit drug use was a risk factor (ORaj = 2.56, 95%CI 1.02-6.12). The number of previous treatments was a protective factor for death (ORaj = 0.41, 95% CI 0.18-0.90), while the associated risk factors were diagnosis in emergency services or during hospitalization (ORaj = 2.88; 95%CI 1.28-6.33) and illicit drug use (ORaj = 2.06; 95%CI 1.36-5.59). Finally, the treatment failure outcome presented a protection association only with the number of treatments to which the patient was submitted (ORaj = 0.06; 95% CI 0.03-0.11), similarly to the other unfavorable outcomes analyzed.

**Table 3:** Results of the logistic regression for the abandonment, death by tuberculosis and treatment failure, with class of cure reference, in the treatment of multidrug-resistant tuberculosis, São Paulo, 2006-2015.

Explanatory variables	Cure/abandonment		Death due to tuberculosis		Cure/treatment failure	
		
	OR (95%CI)	ORaj (IC 95%)^1^	OR (95%CI)	ORaj (IC95%)^2^	OR (95%CI)	ORaj (IC95%)^3^
Number of previous MDR treatments						
None	1	1	1	1	1	1
One or more	0.37 (0.18–0.72)	0.33 (0.16–0.66)	0.38 (0.17–0.79)	0.41 (0.18–0.90)	0.05 (0.03–0.10)	0.06 (0.03–0.11)
Race/color						
White	1		1		1	
Black/brown	1.78 (0.91–3.49)		1.16 (0.55–2.40)		0.93 (0.62–1.37)	
Age (years old)						
≤ 40	1		1		1	
> 40	0.67 (0.33–1.31)		1.25 (0.60–2.63)		0.83 (0.56–1.22)	
Gender (n = 856)						
Male	1		1		1	
Female	0.79 (0.37–1.60)		0.61 (0.24–1.36)		0.86 (0.56–1.31)	
Education level						
≤ 7 years	1		1		1	
> 7 years	0.74 (0.37–1.44)		0.61(0.28–1.27)		0.89(0.60–1.31)	
Clinical Form						
Pulmonary	1		1		1	
Extrapulmonary	2.59 (0.11–27.72)		6.65 (0.77–57.08)		1.72 (0.28–13.20)	
Way of discovering						
Outpatient demand and active search	1		1	1	1	1
Urgency/emergency or during hospital stay	1.70 (0.76–3.62)		3.36 (1.53–7.24)	2.88 (1.28–6.33)	2.01 (1.26–3.22)	1.20 (0.69–2.11)
HIV						
Negative	1		1		1	
Positive	0.20 (0.01–1.01)		0.25 (0.01–1.26)		0.54 (0.25–1.09)	
Diabetes						
No	1		1		1	
Yes	1.54 (0.56–5.40)		1.59 (0.59–3.83)		0.82 (0.46–1.47)	
Alcoholism						
No	1		1		1	
Yes	1.79 (0.83–3.68)		1.91 (0.84–4.15)		0.93 (0.57–1.50)	
Mental illness						
No	1		1		1	
Yes	2.63 (0.35–13.98)		1.59 (0.08–11.22)		1.47 (0.26–4.91)	
Use of illicit drugs						
No	1	1	1	1	1	
Yes	2.07 (1.03–4.71)	2.56 (1.02–6.12)	1.62 (1.25–4.10)	2.06 (1.36–5.59)	0.85 (0.45–1.60)	
Smoking						
No	1		1		1	1
Yes	0.56 (0.16–1.51)		1.14 (0.40–2.80)		0.35 (0.17–0.68)	0.52 (0.23–1.14)
Type of address						
Default Address	1		1		1	
Other	1.02 (0.22–3.29)		0.83 (0.12–3.14)		1.07 (0.49–2.29)	
Treatment type						
Supervised	1		1		1	1
Self-administered	1.59 (0.59–3.81)		1.70 (0.59–4.31)		2.34 (1.37–4.09)	1.45 (0.77-2.81)

MDR: multidrug-resistant; OR: odds ratio; ORaj: adjusted odds ratio

^a^ AIC: 222.47; pseudo R^2^ (McFadden): 0.05; Wald: F = 5.87 (p < 0.01); ROC: 0.65 (95%CI 0.57-0.73).

^b^ AIC: 191.62; pseudo R^2^ (McFadden): 0.07; Wald: F = 4.81 (p < 0.01); ROC: 0.70 (95%CI 0.61-0.79).

^c^ AIC: 434.14; pseudo R^2^ (McFadden): 0.26; Wald: F = 26.48 (p < 0.01); ROC: 0.80 (95%CI 0.76-0.84).

The logistic model for abandonment presented an area below the ROC curve of 0.65 (95%CI 0.57-0.73), while the ROC value for the death model was 0.70 (95%CI 0.61-0.79), and for treatment failure, 0.80 (95%CI 0.76-0.84). The discrimination power of the model was classified as “sufficient” to identify the abandonment, “good” to point to death and “very good” for treatment failure. In addition, the diagnosis of the regression models indicated adequacy and non-violation of assumptions.

**Figure 1:**
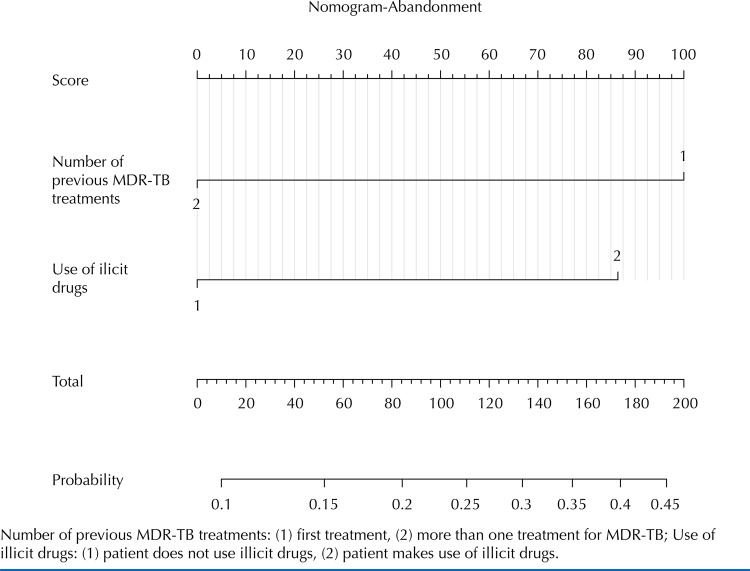
Nomograms of dropouts in the treatment of multidrug-resistant tuberculosis.

**Figure 2:**
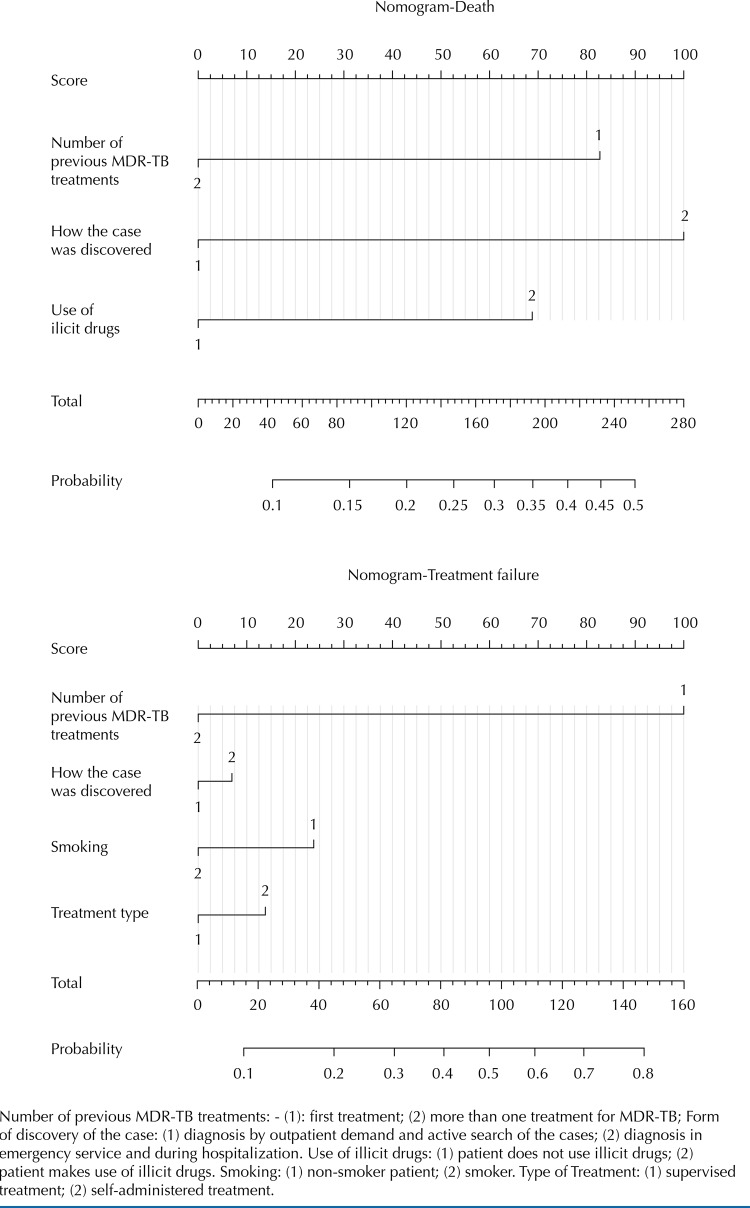
Nomograms of deaths and bankruptcies in the treatment of multidrug-resistant tuberculosis.

The explanatory variables related to the unfavorable outcomes, as indicated in the binary logistic model, were analyzed using the nomogram, which predicts the probability that each characteristic has for abandonment (Figure 1), death and treatment failure (Figure 2). Depending on the individual aspects of the patient in relation to the inserted variables, a score is computed, ranging from zero to 100. After considering all attributes, the patient’s total score is added, which represents the probability of occurrence of the event in question. Thus, it is simplified the understanding of the prediction factor of these characteristics for the unfavorable outcome in the treatment of MDR-TB.

Number of previous MDR-TB treatments: (1) first treatment, (2) more than one treatment for MDR-TB; Use of illicit drugs: (1) patient does not use illicit drugs, (2) patient makes use of illicit drugs.

Number of previous MDR-TB treatments: - (1): first treatment; (2) more than one treatment for MDR-TB; Form of discovery of the case: (1) diagnosis by outpatient demand and active search of the cases; (2) diagnosis in emergency service and during hospitalization. Use of illicit drugs: (1) patient does not use illicit drugs; (2) patient makes use of illicit drugs. Smoking: (1) non-smoker patient; (2) smoker. Type of Treatment: (1) supervised treatment; (2) self-administered treatment.

In the case of abandonment, considering the two variables included in the nomogram, a probability of 20% to 25% was observed for patients treated as MDR-TB virgin and approximately 20% for illicit drug users. Patients who belonged to both categories may present a 40% to 45% probability of dropping out of treatment.

Regarding death, patients on the first treatment of MDR-TB had approximately a 15% chance of death. Those who were diagnosed in emergency services or with elucidation of the case during hospital stay had between 15% and 20% chance. In the case of illicit drug use, the chance was 10% to 15%. Combining these factors, the chance for this unfavorable outcome reached the range of 45% to 50%.

Regarding treatment failure, the only variable with significant association was the number of previous treatments. For those in the first treatment for MDR-TB, there was little more than a 50% likelihood for failure outcome.

## DISCUSSION

The study identified the main risk factors related to death, abandonment and treatment failure in MDR-TB treatments, with evidence of association of these outcomes with illicit drug use, number of previous MDR-TB treatments and place of diagnosis of the case. In addition, the predictive capacity for each of these variables was verified, allowing the identification of individuals at greater risk for unfavorable outcomes.

The results showed that in the state of São Paulo there was a growth in the proportion of patients’ cure and a trend to reduce treatment failure, highlighting a visible conversion of these outcomes mainly from 2011, something also observed by Valdes et al.^10^. These trends are presumed to be related to the application of norms for diagnosis and treatment published in 2007, which allowed over time to reverse the state scenario of MDR-TB treatment observed between the 2006 and 2010 and to incorporate new drugs and regimens treatment in the state^11^. In addition, health teams have possibly accumulated experience and knowledge about the disease and care of these patients to the point of improve the outcome of cases. However, it is important to note that the proportion of cures in 2015, when the highest value (59.38%) of the study period was observed, falls short of the goals established by WHO.

Regarding the outcomes of treatment abandonment and death with TB, both showed stationary trends, indicating that they are still key elements for the control of unfavorable outcomes of MDR-TB. Thus, it is important to strengthen both TB control programs and health services in the management and follow-up of these patients, especially considering that the reasons for patients discontinuing treatment and developing more severe forms of the disease are usually related to psychosocial factors.

Retreatment of MDR-TB was presented as a protective factor for outcomes, an unexpected result. A study by Bastos et al.^12^ in Brazil identified that the episode of resistant TB being the first in the patient’s life resulted in higher chances of a positive outcome. Possibly, the divergence of these results with this study is in the agglutination of cases with mono and multiresistance in the same study population and in the fact that the Brazilian Southeast presents the worst results for cure, being able to represent a specific and distinct cohort of the rest of the country.

In addition, in China^13^ and Pakistan^14^ studies, there was a tendency for patients with previous unfavorable outcomes to have a lower chance of repeating them, even though such outcomes have not been significantly demonstrated in multivariate analyzes. In addition, failure to complete the “entry-type” field on the patient notification form on TBWeb made it impossible to identify whether patients who underwent new MDR-TB treatments did so due to abandonment, treatment failure, or relapse.

Despite the need to cautiously observe the previous treatments for MDR-TB and its relation to the outcomes, there is a possible explanation for the higher success rate in individuals who have already undergone it. After the first treatment, patients are presumably assisted more effectively by the health services and their professionals, who, considering previous outcomes, carry out educational actions on the disease, advising on the importance of ending the treatment, in addition to providing psychosocial support to both the patient and family members^15^. It cannot be disregarded the effect that failure to achieve cure has on the perception of threat of death for the patient, which may influence self-regulation and motivation to end the therapeutic regimen^15^.

In addition, previously treated and negatively evolving patients should be examined more rigorously before starting new treatments as recommended by PNCTB^6^, identifying the profile of susceptibility to second-line drugs more effectively and listing drug combinations for MDR-TB with a lower chance of treatment failure^16,17^.

However, it is important to consider the repercussions of repeated treatments for patients and for the health system itself. Despite universal access to medication, therapy usually implies high costs of transportation and food, resulting in loss of family income and catastrophic expenses^18^. For the health system, costs are also high, and the need for more than one treatment for therapeutic success can significantly burden public administration^19^. In addition, there are several sequelae of MDR-TB treatment, which can be aggravated by multiple therapeutic cycles. Some studies indicate that 90% of the treated cases present some sequelae, the most common being pulmonary dysfunctions such as dyspnea and reduced vital capacity, hearing loss due to ototoxicity, loss of quality of life and social isolation^20^.

Another risk factor specifically related to dropout and death from MDR-TB was the use of illicit drugs. This evidence has already been presented in other studies, both for cases of sensitive TB^21^ and for the resistant form of the disease^22^. It should be noted that illicit drug users are considered patients of great complexity, both due to the difficulty in preventing infection and developing the active form of the disease, as well as the comorbidities commonly associated with cases such as hepatitis B, hepatitis C and or HIV^23^. Such a population is afflicted by a high burden of stigma of TB, of illegal drug use and of a chance of an illicit lifestyle, as reported by Hayashi et al.^24^. These authors identified that 80% of injecting drug users had already been incarcerated in prisons at least once. As a consequence, they move away from the care provided by health professionals, and adherence to treatment is compromised to the point that services are unable to reach contact and bond^25^.

Based on these considerations and the results presented in the nomogram, it can be hypothesized that approximately half of the illicit drug users who are on the first MDR-TB treatment will abandon it and one-fourth may become fatal. Thus, for MDR-TB treatment of illicit drug users to have better results, it is necessary that specific measures are developed to ensure their full care^22^.

The health service in which the diagnosis was made showed an association with death due to MDR-TB. Patients whose case was elucidated in emergency services in the hospital network showed more than double the chance of death due to the disease.

Although primary health care (PHC) is recognized as the preferred entry point and structuring axis of the health system and is considered a priority for the control and care of patients with TB^26^, more than 25% of MDR-TB cases in the state of São Paulo were diagnosed in other types of services, evidencing the difficulty of PHC in carrying out the timely diagnosis of the disease. The reasons for the population to seek other ports of entry may be related to the difficulty of access to PHC, both due to the restricted hours of operation of the units and the low coverage of attention, and the cultural habit of seeking emergency units^27^.

The effects of delayed diagnosis may be extensive, as it results in a greater chance of MDR-TB transmission in the population, of patient suffering and of disease progression, which impairs the treatment outcome and increases the risk of death^28^. In this way, it is necessary to strengthen the actions to search for cases in the community and overcome old-fashioned system policies, expand and consolidate PHC coverage in municipalities, and invest in qualified professionals prepared to find cases appropriately^29^.

The construction of a prognostic model such as the nomogram can mean better therapeutic results and reduction of deaths, dropouts and failures in the treatment of MDR-TB. The nomogram tool is often used in the oncology area and considered simple and effective for a better prognosis of diseases^30^. One of its main advantages is the ability to individually estimate patients’ risk based on their own characteristics, helping in decision making^9^.

Considering the discrimination capacity of the logistic models of this study, it is possible to state that the nomogram presented has the capacity to predict the occurrence of abandonment, death and treatment failure in 65%, 70% and 80% of the cases, respectively. However, it is important to understand that these results have internal validation in the studied cohort and that other scenarios require a validation process for the population under analysis.

The study has limitations related to its retrospective character, meaning the inability to control the data collected from the TBWeb information system. In addition, the data may suffer from underreporting, mainly due to the diagnostic difficulty involved in MDR-TB, caused both by the lack of indication of patients to sensitivity tests and by the difficulty of access to technological equipment such as GeneXpert for all municipalities in the state of Sao Paulo.
