# Supplementary material for: Predictive model of unfavorable outcomes for multidrug-resistant tuberculosis
Source: Rev Saude Publica. 2019 Sep 12;53:77. doi: 10.11606/s1518-8787.2019053001151 (PMC6752648; doi:10.11606/s1518-8787.2019053001151)
Supplement: Translation

# Translation: Modelo preditivo dos desfechos desfavoráveis da tuberculose multidroga-resistente

**Authors:** Luiz Henrique Arroyo, Antônio Carlos Vieira Ramos, Mellina Yamamura, Thais Zamboni Berra, Luana Seles Alves, Aylana de Souza Belchior, Danielle Talita Santos, Josilene Dália Alves, Laura Terenciani Campoy, Marcos Augusto Moraes Arcoverde, Valdes Roberto Bollela, Sidney Bombarda, Carla Nunes, Ricardo Alexandre Arcêncio

**Affiliations:** I Universidade de São Paulo. Escola de Enfermagem de Ribeirão Preto. Ribeirão Preto, SP, Brasil; II Universidade de São Paulo. Faculdade de Medicina de Ribeirão Preto. Ribeirão Preto, SP, Brasil; III Secretaria de Estado da Saúde de São Paulo. São Paulo, SP, Brasil; IV Universidade NOVA de Lisboa. Escola Nacional de Saúde Pública. Lisboa, Portugal

**Keywords:** Tuberculose Resistente a Múltiplos Medicamentos, complicações, Tuberculose Resistente a Múltiplos Medicamentos, mortalidade, Fatores de Risco, Cooperação e Adesão ao Tratamento

## Abstract

**OBJETIVO:**

Analisar a tendência temporal, identificar os fatores relacionados e elaborar um modelo preditivo para os desfechos desfavoráveis do tratamento da tuberculose multidroga-resistente.

**MÉTODOS:**

Estudo de coorte retrospectiva com todos os casos diagnosticados com tuberculose multidroga-resistente entre os anos de 2006 e 2015 no estado de São Paulo. Os dados secundários foram provenientes do sistema estadual de notificações de casos de tuberculose, o TBWeb. Foi realizada a análise de tendência temporal dos desfechos de tratamento por meio da regressão de Prais-Winsten. Para verificar os fatores relacionados com os desfechos desfavoráveis (óbito com tuberculose como causa básica, abandono e falência do tratamento), foi empregada a regressão logística binária. Representações pictóricas dos fatores relacionados ao desfecho do tratamento e sua capacidade prognóstica foram elaboradas por meio de nomogramas.

**RESULTADOS:**

Tanto o abandono como o óbito tiveram tendência temporal estacionária, enquanto a falência apresentou tendência decrescente. Em relação aos fatores de risco para tais desfechos, utilizar drogas ilícitas dobrou as chances de abandono e óbito. Além disso, ser diagnosticado em unidades de urgência ou emergência ou durante internações hospitalares foi um fator de risco para o óbito. Ao contrário, ter feito tratamentos prévios da multidroga-resistência reduziu as chances dos desfechos analisados. O nomograma apresentou um modelo preditivo com precisão de 65% para os abandonos, 70% para os óbitos e 80% para a falência.

**CONCLUSÕES:**

A prevenção de desfechos desfavoráveis no tratamento da tuberculose multidroga-resistente implica a modificação do modelo de atenção vigente. Utilizando modelos preditivos, como o apresentado neste estudo, torna-se possível elaborar ações centradas nos pacientes, considerando seus fatores de risco e aumentando as chances de cura.

## INTRODUÇÃO

A tuberculose multidroga-resistente (TB-MDR) é considerada um problema global de saúde pública e grande ameaça para o controle e eliminação da tuberculose (TB) no mundo. Caracterizada por um bacilo com resistência a isoniazida e rifampicina, dois dos principais fármacos do esquema inicial de tratamento, estima-se que a TB-MDR em 2016 tenha apresentado cerca de 490 mil casos, o equivalente a 4,7% do total de pessoas que adoecerem por TB no mundo^1^.

De acordo com o último relatório da Organização Mundial da Saúde (OMS), dos casos que iniciaram tratamento para a TB-MDR em 2014, 54% o completaram com sucesso (cura ou tratamento completo), 15% abandonaram, 8% tiveram falha e 16% morreram. Comparando aos desfechos dos casos novos ou recidivas da TB em que o bacilo é sensível às drogas de primeira linha, houve 83% de cura, indicando que o aumento dos percentuais de sucesso no tratamento da TB-MDR é um dos principais desafios globais para o controle da doença^1^.

A dificuldade de sucesso é consequência da insuficiência de terapias que possibilitem coordenar tratamentos mais efetivos e com maior capacidade para o desfecho favorável. Os regimes de tratamento ainda são baseados em frágeis evidências científicas, e as drogas utilizadas nem sempre passam por ensaios clínicos randomizados e controlados, o que resulta em uma alta frequência de falências no tratamento^2^.

Ademais, os longos períodos de exposição a drogas com alta toxicidade e que podem desencadear graves efeitos adversos, somados a tratamentos que suscitam gastos catastróficos das famílias, estigma social e estresse psicológico, reduzem a adesão e tolerância dos pacientes ao tratamento da TB-MDR e aumentam o risco para desfechos desfavoráveis como o óbito, abandono e falência^3,4^.

Apesar disso, o país apresenta baixas proporções de cura para o tratamento da TB-MDR, chegando a apenas 61,4%, porcentagem aquém da estabelecida como ideal pela OMS (75%)^1^. Dessa forma, torna-se premente a identificação das barreiras que inviabilizam a cura dos pacientes, sob pena da transmissão do *Mycobacterium tuberculosis* resistente, o que contribui para o aumento da proporção da TB-MDR entre os casos incidentes de TB e do desenvolvimento de formas mais graves da doença, como a tuberculose extensivamente resistente (TB-XDR)^5^.

Assim, levando em consideração a complexidade envolvida no sucesso do tratamento da TB-MDR e a escassez de pesquisas acerca da temática, este estudo tem como objetivo caracterizar os desfechos de tratamento de TB-MDR e analisar a tendência temporal e fatores relacionados aos desfechos desfavoráveis no estado de São Paulo.

## MÉTODOS

### Desenho e população do estudo

Estudo de coorte retrospectiva com uso de dados secundários do sistema estadual de notificações de casos de TB, denominado TBWeb. Foram utilizados todos os casos diagnosticados com TB-MDR entre os anos de 2006 e 2015 nos 645 municípios do estado de São Paulo. Os dados foram coletados no Centro de Vigilância Epidemiológica Prof. Alexandre Vranjac em dezembro de 2017.

### Variáveis e análise estatística

Inicialmente, na abordagem exploratória do banco de dados foram retiradas as duplicidades. Nesse processo foram utilizados o nome completo do indivíduo, nome completo da mãe e data de nascimento, permanecendo para as análises apenas o desfecho mais atual.

A análise descritiva considerou o número de tratamentos prévios para a TB-MDR e as características individuais presentes na ficha de notificação de casos do TBWeb: sociodemográficas (sexo, idade, etnia, escolaridade e tipo de endereço), clínicas (forma clínica da TB-MDR e comorbidades associadas) e operacionais (forma de descoberta do caso e tratamento supervisionado ou autoadministrado). É válido destacar que foram considerados desfechos desfavoráveis os dos casos cujo último encerramento apresentava-se como óbito, abandono e falência do tratamento.

Os desfechos desfavoráveis no tratamento da TB-MDR são estabelecidos pelo Programa Nacional de Controle da Tuberculose (PNCTB) como tratamentos que evoluem de forma negativa e resultam em abandonos, falências ou óbitos. O abandono é definido como a não tomada da medicação por mais de 30 dias consecutivos. A falência do tratamento é definida por duas ou mais culturas positivas após o 12º mês de tratamento ou de acordo com a avaliação do quadro clínico dos pacientes^6^.

Em seguida, foi verificada a ocorrência anual de cada desfecho, incluindo todos os tipos disponíveis para preenchimento na ficha de notificação (cura, abandono, falha no tratamento, óbito TB, óbito não TB, outros desfechos, sem informação de desfecho). Posteriormente, foram realizadas análises de tendências temporais (regressão temporal), cujas variáveis preditivas foram o número de desfechos de cura, abandono, falha no tratamento, óbito com causa básica TB e óbito sem causa básica TB, e a variável resposta foi o tempo (em anos).

Os desfechos foram logaritmizados, reduzindo a heterogeneidade da variância dos resíduos na regressão temporal. Essa tendência temporal foi realizada usando o método de análise autorregrada conhecido como Prais-Winsten, cujo resultado é denominado taxa de incremento anual, com intervalo de confiança de 95% (IC95%). Os resultados significativos poderiam representar o crescimento ou decréscimo anual da ocorrência dos desfechos, enquanto os resultados não significativos podem ser considerados estacionários^7^.

Para verificar os fatores relacionados com os desfechos desfavoráveis, foi empregada a regressão logística binária tendo como referência os tratamentos com cura e como variáveis independentes as informações individuais dos pacientes. Ressalta-se que foram utilizados modelos distintos para cada um dos desfechos.

Na primeira etapa, foi calculado o *odds ratio* bruto (OR) com IC95%. Subsequentemente, as variáveis com OR significativo foram incluídas no modelo múltiplo com método *forward (likelihood ratio)*, determinando seu *odds ratio* ajustado (ORaj). Para ambos os modelos finais, foram calculados os pseudocoeficientes de determinação (R^2^ de McFadden), estatística de Wald e capacidade de predição ou acurácia dos modelos utilizando a área abaixo da curva de característica de operação do receptor ou *receiver operating characteristic* (ROC) e seus respectivos valores de IC95%. Os valores da curva ROC foram interpretadas segundo Šimundić^8^.

Foram feitas representações pictóricas dos fatores relacionados ao óbito, abandono e falência no tratamento da TB-MDR e sua capacidade prognóstica para os desfechos em forma de probabilidades. Tal técnica é denominada nomograma e é apresentada como uma escala de pontuação para cada variável introduzida na análise. Essa pontuação é equivalente a uma determinada probabilidade para o evento em estudo, neste caso, os desfechos de tratamento^9^.

### Aspectos Éticos

O estudo foi aprovado pelo Comitê de Ética em Pesquisa da Escola de Enfermagem de Ribeirão Preto da Universidade de São Paulo em 12 de setembro de 2017, com número de protocolo de Certificado de Apresentação para Apreciação Ética (CAAE) 71051017.8.0000.5393.

## RESULTADOS

Foram identificadas 1.168 notificações de MDR-TB no estado de São Paulo no período de 2006 a 2015. Após a filtragem das notificações duplicadas, verificou-se o total de 802 pacientes acometidos pela doença. Suas características sociodemográficas e clínico-operacionais são apresentadas na Tabela 1.

**Tabela 1:** Perfil dos 802 casos de tuberculose multidroga-resistente notificados no estado de São Paulo de 2006 a 2015.

Variável	n	%
Desfecho dos tratamentos		
Cura	323	40,3
Abandono	66	8,2
Falência do tratamento	275	34,3
Mudança do diagnóstico	6	0,7
Óbito não tuberculose	55	6,8
Óbito por tuberculose	56	7,0
Transferência	5	0,7
Sem informações	16	2,0
Número de tratamentos		
Um tratamento	522	65,1
Mais de um tratamento	280	34,9
Etnia		
Branco	358	44,6
Preto/pardo	267	33,3
Outros (amarelo, indígena)	6	0,7
Sem informações	171	21,3
Idade (anos)		
≤ 14	7	0,9
15–29	197	24,5
30–59	532	66,3
≥ 60	66	8,2
Sexo		
Masculino	564	70,3
Feminino	238	29,7
Escolaridade		
≤ 7 anos	354	44,1
> 7 anos	311	38,8
Sem informações	137	17,1
Forma clínica		
Pulmonar	782	97,5
Extrapulmonar	20	2,5
Forma de descoberta		
Demanda ambulatorial	531	66,2
Urgência/emergência ou durante internação hospitalar	205	25,6
Busca ativa de casos	38	4,7
Sem informações	28	3,5
Exame HIV		
Realizado	735	91,6
Não realizado	67	8,4
Aids		
Sim	104	13,0
Não	621	77,4
Sem informação*	77	9,6
Diabetes		
Sim	103	12,8
Não	699	87,2
Alcoolismo		
Sim	183	22,8
Não	619	77,2
Doença mental		
Sim	12	1,5
Não	790	98,5
Uso de drogas ilícitas		
Sim	96	12,0
Não	706	88,0
Tabagismo		
Sim	70	8,7
Não	732	91,3
Tipo de endereço		
Endereço padrão	742	92,5
Detento	44	5,5
Sem residência fixa	16	3,0
Tipo de tratamento		
Supervisionado	663	76,5
Autoadministrado	154	17,7
Sem informação	50	5,8

* Foram considerados sem informação 10 casos com teste HIV positivo, mas não classificados com aids.

A cura foi o desfecho de tratamento com maior predominância (n = 323; 40,3%); contudo, a falência do tratamento foi quase tão frequente, com 275 (34,3%) ocorrências. Outros desfechos desfavoráveis, como abandono e óbito com causa básica TB, ocorreram em 15,2% (n = 122) dos casos. Apesar da predominância de pacientes submetidos a apenas um tratamento, 34,9% (n = 280) já tinham sido tratados para TB-MDR anteriormente.

No que se refere ao perfil sociodemográfico, houve predominância de casos do sexo masculino, com idade entre 15 e 59 anos, brancos e com menos de sete anos de escolaridade. Em relação às características clínico-operacionais, prevaleceu a forma pulmonar da doença e o diagnóstico foi realizado em geral por demandas ambulatoriais; todavia, um quarto dos casos foi descoberto em serviços de urgência, emergência ou durante a internação hospitalar (n = 205; 25,6%).

Nenhuma das comorbidades registradas se mostrou com maior prevalência entre os casos; não obstante, a comorbidade de maior frequência foi o alcoolismo, seguido pela HIV/aids e diabetes. A maioria dos pacientes registrados tinha endereço padrão (n = 742; 92,5%), com os indivíduos privados de liberdade formando 5,5% (n = 44) da coorte e o restante representando pessoas sem residência fixa.

Observando os desfechos do tratamento por ano estudado (Tabela 2), constata-se que a taxa média de cura entre os anos de 2006 e 2010 foi de 25,77% e de 55,22% nos anos subsequentes. A análise de tendência temporal demonstrou o crescimento da proporção de cura em 5% ao ano. A falência no tratamento foi o desfecho predominante entre os primeiros quatro anos estudados; todavia, após 2011 essa porcentagem apresentou queda, atingindo apenas 7,29% em 2015. Na interpretação da tendência temporal, esse desfecho decresceu na ordem de 8% ao ano. Ademais, o abandono e os óbitos por TB mantiveram-se praticamente constantes nos anos observados, o que foi confirmado pela tendência estacionária dos casos.

**Tabela 2:** Distribuição de desfechos de tratamento por ano e tendência temporal, São Paulo, 2006–2015.

Desfecho	2006	2007	2008	2009	2010	2011	2012	2013	2014	2015	Total	Coeficiente	IC95%	Tendência temporal
									
	n (%)	n (%)	n (%)	n (%)	n (%)	n (%)	n (%)	n (%)	n (%)	n (%)				
Cura	28 (34,57)	18 (26,87)	20 (29,85)	14 (14,58)	20,00 (22,99)	47 (50,54)	42 (54,55)	33 (55,93)	44 (55,70)	57 (59,38)	323 (40,27)	0,05	0,01–0,09	Crescente
Abandono	4 (4,94)	4 (5,97)	7 (10,45)	12 (12,50)	5,00 (5,75)	4 (4,30)	5 (6,49)	6 (10,17)	7 (8,86)	12 (12,50)	66 (8,23)	0,03	-0,02–0,08	Estacionário
Falha no tratamento	37 (45,68)	34 (50,75)	33 (49,25)	52 (54,17)	42,00 (48,28)	30 (32,26)	17 (22,08)	12 (20,34)	11 (13,92)	7 (7,29)	275 (34,29)	-0,08	-0,01– -0,02	Decrescente
Óbito TB	3 (3,70)	7 (10,45)	1 (1,49)	8 (8,33)	8 (9,19)	4 (4,30)	5 (6,49)	5 (8,47)	7 (8,86)	8 (8,33)	56 (6,98)	0,03	-0,01–0,08	Estacionário
Óbito não TB	6 (7,41)	2 (2,98)	4 (5,97)	9 (9,37)	10 (11,49)	5 (5,38)	7 (9,09)	3 (5,08)	6 (7,59)	3 (3,12)	55 (6,86)	-0,01	-0,06–0,05	Estacionário
Outros	2 (2,47)	1 (1,49)	0 (0)	0 (0,00)	2 (2,30)	2 (2,15)	1 (1,30)	0 (0)	1 (1,27)	2 (2,08)	11 (1,38)	-	-	-
Sem informação	1 (1,23)	1 (1,49)	2 (2,99)	1 (1,04)	0 (0)	1 (1,08)	0 (0,00)	0 (0)	3 (3,80)	7 (7,29)	16 (1,99)	-	-	-

Total	81 (10,10)	67 (8,35)	67 (8,35)	96 (11,97)	87 (10,85)	93 (11,60)	77 (9,60)	59 (7,36)	79 (9,85)	96 (11,97)	802 (100)	<0,01	-0,01–0,02	Estacionário

TB: tuberculose

Na análise dos fatores associados aos desfechos desfavoráveis na coorte estudada (Tabela 3), são apresentados os valores de OR bruto e ajustado para óbito com causa básica TB, abandono e falência do tratamento. O histórico de um ou mais tratamentos prévios de TB-MDR (ORaj = 0,33; IC95% 0,16–0,66) foi fator de proteção para o abandono, enquanto o uso de drogas ilícitas foi fator de risco (ORaj = 2,56; IC95% 1,02–6,12). O número de tratamentos prévios apresentou-se como fator protetor para o óbito (ORaj = 0,41; IC95% 0,18–0,90), enquanto os fatores de risco associados foram o diagnóstico em serviços de urgência, emergência ou durante internação hospitalar (ORaj = 2,88; IC95% 1,28–6,33) e uso de drogas ilícitas (ORaj = 2,06; IC95% 1,36–5,59). Por fim, o desfecho falência apresentou associação de proteção apenas com o número de tratamentos a que o paciente foi submetido (ORaj = 0,06; IC95% 0,03–0,11), de forma semelhante aos outros desfechos desfavoráveis analisados.

**Tabela 3:** Resultados da regressão logística para o abandono, óbito por tuberculose e falência, com classe de referência cura, no tratamento da tuberculose multidroga-resistente, São Paulo, 2006–2015.

Variáveis explicativas	Cura/abandono		Cura/óbito por tuberculose		Cura/falência	
		
	OR (IC95%)	ORaj (IC95%)^1^	OR (IC95%)	ORaj (IC95%)^2^	OR (IC95%)	ORaj (IC95%)^3^
Número de tratamentos MDR prévios						
Nenhum	1	1	1	1	1	1
Um ou mais	0,37 (0,18–0,72)	0,33 (0,16–0,66)	0,38 (0,17–0,79)	0,41 (0,18–0,90)	0,05 (0,03–0,10)	0,06 (0,03–0,11)
Raça/cor						
Branco	1		1		1	
Preto/pardo	1,78 (0,91–3,49)		1,16 (0,55–2,40)		0,93 (0,62–1,37)	
Idade (anos)						
≤ 40	1		1		1	
> 40	0,67 (0,33–1,31)		1,25 (0,60–2,63)		0,83 (0,56–1,22)	
Sexo (n = 856)						
Masculino	1		1		1	
Feminino	0,79 (0,37–1,60)		0,61 (0,24–1,36)		0,86 (0,56–1,31)	
Escolaridade						
≤ 7 anos	1		1		1	
> 7 anos	0,74 (0,37–1,44)		0,61(0,28–1,27)		0,89(0,60–1,31)	
Forma clínica						
Pulmonar	1		1		1	
Extrapulmonar	2,59 (0,11–27,72)		6,65 (0,77–57,08)		1,72 (0,28–13,20)	
Forma de descoberta						
Demanda ambulatorial e busca ativa	1		1	1	1	1
Urgência/emergência ou durante internação hospitalar	1,70 (0,76–3,62)		3,36 (1,53–7,24)	2,88 (1,28–6,33)	2,01 (1,26–3,22)	1,20 (0,69–2,11)
HIV						
Negativo	1		1		1	
Positivo	0,20 (0,01–1,01)		0,25 (0,01–1,26)		0,54 (0,25–1,09)	
Diabetes						
Não	1		1		1	
Sim	1,54 (0,56–5,40)		1,59 (0,59–3,83)		0,82 (0,46–1,47)	
Alcoolismo						
Não	1		1		1	
Sim	1,79 (0,83–3,68)		1,91 (0,84–4,15)		0,93 (0,57–1,50)	
Doença mental						
Não	1		1		1	
Sim	2,63 (0,35–13,98)		1,59 (0,08–11,22)		1,47 (0,26–4,91)	
Uso de drogas ilícitas						
Não	1	1	1	1	1	
Sim	2,07 (1,03–4,71)	2,56 (1,02–6,12)	1,62 (1,25–4,10)	2,06 (1,36–5,59)	0,85 (0,45–1,60)	
Tabagismo						
Não	1		1		1	1
Sim	0,56 (0,16–1,51)		1,14 (0,40–2,80)		0,35 (0,17–0,68)	0,52 (0,23–1,14)
Tipo de endereço						
Endereço padrão	1		1		1	
Outros	1,02 (0,22–3,29)		0,83 (0,12–3,14)		1,07 (0,49–2,29)	
Tipo de tratamento						
Supervisionado	1		1		1	1
Autoadministrado	1,59 (0,59–3,81)		1,70 (0,59–4,31)		2,34 (1,37–4,09)	1,45 (0,77-2,81)

MDR: multidroga-resistente; OR: *odds ratio*; ORaj: *odds ratio* ajustado

^a^ AIC: 222,47; pseudo R^2^ (McFadden): 0,05; Wald: F = 5,87 (p < 0,01); ROC: 0,65 (IC95% 0,57–0,73).

^b^ AIC: 191,62; pseudo R^2^ (McFadden): 0,07; Wald: F = 4,81 (p < 0,01); ROC: 0,70 (IC95% 0,61–0,79).

^c^ AIC: 434,14; pseudo R^2^ (McFadden): 0,26; Wald: F = 26,48 (p < 0,01); ROC: 0,80 (IC95% 0,76–0,84).

O modelo logístico para o abandono apresentou área abaixo da curva ROC de 0,65 (IC95% 0,57–0,73), enquanto o valor ROC para o modelo óbito foi igual a 0,70 (IC95% 0,61–0,79) e para a falência, 0,80 (IC95% 0,76–0,84). O poder de discriminação do modelo foi classificado como “suficiente” para identificar o abandono, “bom” para apontar o óbito e “muito bom” para a falência. Ademais, o diagnóstico dos modelos de regressão indicou adequação e não violação de pressupostos.

As variáveis explicativas relacionadas com os desfechos desfavoráveis, conforme apontado no modelo logístico binário, foram analisadas utilizando o nomograma, que prediz a probabilidade que cada característica possui tanto para o abandono (Figura 1) como para o óbito e a falência (Figura 2). Dependendo dos aspectos individuais do paciente em relação às variáveis inseridas, uma pontuação é computada, variando de zero a 100. Após considerar todos os atributos, soma-se a pontuação total do paciente, valor que representa a probabilidade de ocorrência do evento em questão. Dessa forma, simplifica-se a compreensão do fator de predição dessas características para o desfecho desfavorável no tratamento da TB-MDR.

**Figura 1:** Nomogramas dos abandonos no tratamento da tuberculose multidroga-resistente

Número de tratamentos TB-MDR prévios: (1) primeiro tratamento; (2) mais de um tratamento para TB-MDR; Uso de drogas ilícitas: (1) paciente não utiliza drogas ilícitas; (2) paciente faz uso de drogas ilícitas.

**Figura 2:** Nomogramas dos óbitos e falências no tratamento da tuberculose multidroga-resistente.

Número de tratamentos TB-MDR prévios: – (1): primeiro tratamento; (2) mais de um tratamento para TB-MDR; Forma de descoberta do caso: (1) diagnóstico por demanda ambulatorial e busca ativa dos casos; (2) diagnóstico em serviço de urgência, emergência e durante internação hospitalar; Uso de drogas ilícitas: (1) paciente não utiliza drogas ilícitas; (2) paciente faz uso de drogas ilícitas; Tabagismo: (1) paciente não tabagista; (2) paciente tabagista; Tipo de Tratamento: (1) tratamento supervisionado; (2) tratamento autoadministrado.

No caso do abandono, levando em consideração isoladamente as duas variáveis inseridas no nomograma, verificou-se uma probabilidade de 20% a 25% para pacientes tidos como virgens de tratamento para TB-MDR e de aproximadamente 20% para os usuários de drogas ilícitas. Pacientes que pertenciam a ambas as categorias podem apresentar uma probabilidade de 40% a 45% de abandono do tratamento.

Em relação ao óbito, pacientes em primeiro tratamento da TB-MDR apresentaram aproximadamente 15% de chance de óbito. Aqueles que foram diagnosticados em serviços de urgência, emergência ou com elucidação do caso durante internação hospitalar tiveram entre 15% e 20% de chance. No caso do uso de drogas ilícitas, a chance era de 10% a 15%. Unindo esses fatores, a chance para esse desfecho desfavorável alcançou o intervalo de 45% a 50%.

No tocante à falência, a única variável com associação significativa foi o número de tratamentos prévios. Para aqueles no primeiro tratamento para TB-MDR, houve pouco mais que 50% de probabilidade para o desfecho falência.

## DISCUSSÃO

O estudo identificou os principais fatores de risco relacionados com o óbito, abandono e falência nos tratamentos para a TB-MDR, com evidências de associação desses desfechos ao uso de drogas ilícitas, número de tratamentos prévios de TB-MDR e local de diagnóstico do caso. Além disso, foi verificada a capacidade preditiva para cada uma dessas variáveis, propiciando a identificação de indivíduos em maior risco para desfechos desfavoráveis.

Os resultados mostraram que no estado de São Paulo houve um crescimento na proporção de cura dos pacientes e uma tendência de redução da falência no tratamento, destacando uma visível conversão desses desfechos principalmente a partir de 2011, algo também observado por Valdes et al.^10^. Presume-se que tais tendências estejam relacionadas à aplicação de normas para o diagnóstico e tratamento publicadas em 2007, que possibilitaram com o tempo reverter o cenário estadual de tratamento da TB-MDR observado entre os anos de 2006 e 2010 e incorporar novas drogas e esquemas de tratamento mais efetivos no estado^11^. Além disso, as equipes de saúde possivelmente acumularam experiências e conhecimento acerca da doença e do cuidado desses pacientes a ponto de atender os casos com maior resolutividade. Todavia, é importante destacar que a proporção de curas em 2015, ano em que se observou o maior valor (59,38%) do período estudado, está aquém das metas estabelecidas pela OMS.

No que concerne aos desfechos abandono do tratamento e óbito com causa TB, ambos demonstraram tendências estacionárias, indicando que ainda são elementos-chave para o controle dos desfechos desfavoráveis da TB-MDR. Dessa forma, é importante fortalecer tanto os programas de controle da TB como os serviços de saúde no manejo e acompanhamento desses pacientes, especialmente considerando que as razões para os pacientes interromperem o tratamento e desenvolverem formas mais graves da doença normalmente estão relacionadas a fatores psicossociais.

O retratamento da TB-MDR se apresentou como um fator protetor para os desfechos, um resultado inesperado. Um estudo elaborado por Bastos et al.^12^ no Brasil identificou que o episódio de TB resistente ser o primeiro na vida do paciente resultou em maiores chances de um desfecho positivo. Possivelmente, a divergência desses resultados com o presente estudo está na aglutinação de casos com mono e multirresistência em uma mesma população de estudo e no fato de o Sudeste brasileiro apresentar os piores resultados para a cura, podendo representar uma coorte específica e distinta do restante do país.

Ademais, em pesquisas da China^13^ e Paquistão^14^, houve uma tendência de os pacientes com desfechos desfavoráveis anteriores apresentarem menor chance de repeti-los, mesmo que tais resultados não tenham sido comprovados significativamente nas análises multivariadas. Somado a isso, a falta de preenchimento do campo “tipo de entrada” na ficha de notificação dos pacientes no TBWeb impossibilitou identificar se os pacientes que realizaram novos tratamentos para TB-MDR o fizeram devido a abandono, falência ou recidiva.

Apesar da necessidade de observar com cautela os tratamentos prévios para TB-MDR e sua relação com os desfechos, há uma possível explicação para a maior taxa de sucesso em indivíduos que já foram submetidos a eles. Após o primeiro tratamento, os pacientes presumivelmente são assistidos de forma mais efetiva pelos serviços de saúde e seus profissionais, que, atentos aos desfechos anteriores, realizam ações de cunho educativo sobre a doença, aconselhando sobre a importância de finalizar o tratamento, além de prover suporte psicossocial tanto ao paciente como aos familiares^15^. Não se pode desconsiderar o efeito que o insucesso de alcançar a cura tem na percepção de ameaça de morte para o paciente, o que pode influir na autorregulação e motivação para finalizar o regime terapêutico^15^.

Além disso, os pacientes previamente tratados e que evoluíram negativamente devem ser examinados com maior rigor antes de iniciar novos tratamentos, como preconizado pelo próprio PNCTB^6^, identificando o perfil de suscetibilidade às drogas de segunda linha de forma mais efetiva e elencando associações medicamentosas para a TB-MDR com menor chance de falência do tratamento^16,17^.

Entretanto, é importante considerar as repercussões dos tratamentos repetidos para os pacientes e para o próprio sistema de saúde. Apesar do acesso universal aos medicamentos, a terapia geralmente implica em altos custos de transporte e alimentação, resultando em perda de renda familiar e gastos catastróficos^18^. Para o sistema de saúde, os custos também são elevados, e a necessidade de mais de um tratamento para o sucesso terapêutico pode onerar significativamente a administração pública^19^. Além disso, há diversas sequelas do tratamento da TB-MDR, que podem ser agravadas pelos múltiplos ciclos terapêuticos. Alguns estudos apontam que 90% dos casos tratados apresentam alguma sequela, as mais comuns sendo disfunções pulmonares como dispneia e redução da capacidade vital, perdas auditivas devido à ototoxicidade, perda da qualidade de vida e isolamento social^20^.

Outro fator de risco relacionado especificamente ao abandono e ao óbito pela TB-MDR foi o uso de drogas ilícitas. Essa evidência já havia sido apresentada em outros estudos, tanto para casos de TB sensível^21^ como para a forma resistente da doença^22^. Há de se destacar que os usuários de drogas ilícitas são considerados pacientes de grande complexidade, tanto pela dificuldade de prevenção da infecção e desenvolvimento da forma ativa da doença, como também pelas comorbidades comumente associadas aos casos, como a hepatite B, a hepatite C e o HIV^23^. Tal população é afligida por uma alta carga de estigma, tanto pela TB, como pelo consumo de drogas consideradas ilegais e pela chance de uma vida de ilicitudes, como relatado por Hayashi et al.^24^. Esses autores identificaram que 80% dos pacientes que utilizavam drogas injetáveis já haviam sido encarcerados em unidades prisionais pelo menos uma vez. Em consequência, eles se afastam do cuidado prestado pelos profissionais de saúde, e a aderência ao tratamento é comprometida a ponto de os serviços não conseguirem contato e vínculo^25^.

A partir dessas considerações e dos resultados apresentados no nomograma, pode-se conjecturar que aproximadamente metade dos pacientes usuários de drogas ilícitas e que estão no primeiro tratamento para TB-MDR irá abandoná-lo e um quarto poderá evoluir para o óbito. Assim, para que o tratamento da TB-MDR em usuários de drogas ilícitas tenha melhores resultados, é necessário que medidas específicas sejam elaboradas para garantir seu cuidado integral^22^.

O serviço de saúde no qual foi realizado o diagnóstico apresentou associação com o óbito por TB-MDR. Pacientes cujo caso foi elucidado em serviços de urgência, emergência ou na rede hospitalar apresentaram mais que o dobro de chance de ir a óbito pela doença.

Apesar de a atenção primária à saúde (APS) ser reconhecida como porta de entrada preferencial e eixo estruturante do sistema de saúde e considerada prioritária para o controle e cuidado dos pacientes de TB^26^, mais de 25% dos casos de TB-MDR no estado de São Paulo foram diagnosticados em outros tipos de serviços, evidenciando a dificuldade da APS em desempenhar o diagnóstico oportuno da doença. Os motivos para a população buscar outras portas de entrada podem estar relacionados à dificuldade de acesso à APS, tanto pelos horários restritos de funcionamento das unidades como pela baixa cobertura de atenção, e ao próprio hábito cultural de buscar unidades de urgência e emergência^27^.

Os efeitos do atraso no diagnóstico podem ser extensos, pois ele resulta em maiores chances de transmissão da TB-MDR na população, de sofrimento dos pacientes e de progressão da doença, que prejudica o desfecho do tratamento e aumenta o risco de morte^28^. Dessa forma, é necessário fortalecer as ações de busca dos casos na comunidade e superar políticas antiquadas do sistema, ampliar e consolidar a cobertura da APS nos municípios e investir em profissionais qualificados e preparados para a descoberta de casos de forma apropriada^29^.

A construção de um modelo prognóstico como o nomograma pode significar melhores resultados terapêuticos e redução dos óbitos, abandonos e falências no tratamento da TB-MDR. A ferramenta do nomograma é utilizada frequentemente na área da oncologia e considerada simples e eficaz para um melhor prognóstico de doenças^30^. Uma das suas principais vantagens é a habilidade de estimar individualmente o risco dos pacientes com base em suas próprias características, auxiliando na tomada de decisões^9^.

Levando em consideração a capacidade de discriminação dos modelos logísticos do presente estudo, por meio da ROC, é possível afirmar que o nomograma apresentado tem capacidade de prever a ocorrência do abandono, óbito e falência em 65%, 70% e 80% dos casos respectivamente. Entretanto, é importante compreender que esses resultados têm validação interna na coorte estudada e que outros cenários requerem um processo de validação para a população em análise.

O estudo tem limitações relacionadas ao seu caráter retrospectivo, significando a impossibilidade de controle dos dados coletados do sistema de informação TBWeb. Além disso, os dados podem sofrer de subnotificação, principalmente pela dificuldade diagnóstica envolvida na TB-MDR, causada tanto pela falta de indicação dos pacientes aos testes de sensibilidade como pela dificuldade de acesso a equipamentos tecnológicos como o GeneXpert para todos os municípios do estado de São Paulo.
